# Diagnosis of single umbilical artery and risk of foetal congenital malformations by prenatal ultrasound: a retrospective study

**DOI:** 10.1186/s12884-024-06375-5

**Published:** 2024-03-12

**Authors:** Junjin Yu, Qingqin Wu, Fanbin Kong, Yan Ning

**Affiliations:** 1 Department of Obstetrics and Gynecology, Women’s Hospital, Zhejiang University School of Medicine, Hangzhou, Zhejiang China; 2 Department of Pharmacy, Dongshan Hospital, Linyi, Shandong China; 3 Department of Ultrasound, Obstetrics and Gynecology Hospital of Fudan University, Shanghai, China; 4 Department of Pathology, Obstetrics and Gynecology Hospital of Fudan University, No. 128, Shenyang Road, Shanghai, 200082 China

**Keywords:** Single umbilical artery, Foetal malformation, Prenatal diagnosis, Ultrasound

## Abstract

**Background:**

Single umbilical artery (SUA) is strongly associated with foetal structural abnormalities; however, the exact pattern of this association has not been described. We aimed to investigate the occurrence of malformations in singleton pregnancies with SUA in China and to study the association between the absent side of the umbilical artery and foetal malformations.

**Methods:**

This was a retrospective study of singleton pregnancies for which routine first-trimester anatomical screening was performed at 11^+ 0^-13^+ 6^ gestational weeks and, if the pregnancy continued, a second-trimester scan was performed at 20^+ 0^-24^+ 0^ weeks. Data were extracted from records at the referral centre, the Obstetrics and Gynecology Hospital of Fudan University, between January 2011 and April 2019 (*n* = 47,894). Using logistic regression, the odds ratios (OR) with 95% confidence intervals (CIs) were calculated for malformations associated with SUA.

**Results:**

The incidence of SUA in our study was 2.0% (970/47,894). Of all foetuses with SUA, 387 (39.9%) had structural malformations. The malformation type varied, with cardiovascular complications being the most common. A robust association was observed between SUA and oesophageal stenosis or atresia (OR: 25.33), followed by cardiovascular (OR: 9.98–24.02), scoliosis (OR: 18.62), genitourinary (OR: 2.45–15.66), and brain malformations (OR: 4.73–9.12). The absence of the left umbilical artery (*n* = 445, 45.9%) was consistent with that of the right umbilical artery (*n* = 431, 44.4%). Furthermore, a significantly higher rate of an absent right than the left umbilical artery (*p*<0.01) was observed in SUA with foetal abnormalities than in SUA with no malformations.

**Conclusions:**

Overall, we observed a higher risk of various specific malformations in foetuses with SUA, and a strong association between SUA and oesophageal stenosis or atresia. The absence of the right umbilical artery was most common in foetuses with SUA and structural malformations. This study provides a reference for ultrasonographers in conducting foetal structural screening for pregnant women with SUA.

## Introduction

Single umbilical artery (SUA), a nonspecific soft ultrasound marker, is the most common umbilical cord abnormality in foetuses [1]. The term isolated single umbilical artery (ISUA) refers to SUA without foetal structural malformations or chromosomal abnormalities. In previous studies, the prevalence of SUA ranged from 0.18 to 3.47%, depending on the population studied [2–6]. Despite these covariances, smoking [7, 8], assisted reproduction [7], velamentous placenta [9], small embryo [10], first pregnancy, and female foetus [11] were considered the risk factors for SUA. Furthermore, the risk of SUA recurrence has been shown to increase when combined with foetal anomalies during the first pregnancy [7].

Previous studies have shown that ISUA is associated with an increased risk of pregnancy-induced hypertension, gestational diabetes, an increased rate of caesarean delivery, emergency caesarean delivery for foetal distress, and preterm delivery [3, 8, 12–14]. ISUA is also related to adverse neonatal outcomes, such as increased incidence of low birth weight, small size for gestational age, lower 1-min Apgar score, increased neonatal intensive care unit (NICU) hospitalisation rate, and longer NICU hospitalisation time [11, 14]. It may also be an independent risk factor for perinatal mortality in small-for-gestational-age neonates [15]. Although the long-term influence of ISUA has not been extensively studied, it has been reported to increase the risk of respiratory morbidity in newborns and urinary tract infections in childhood [16, 17]. However, it does not affect longitudinal physical growth or neurological outcomes in infants [18].

SUA is strongly associated with foetal structural abnormalities, including multiple system abnormalities, irrespective of the presence of foetal chromosomal abnormalities. Cardiovascular, genitourinary, musculoskeletal, and gastrointestinal abnormalities are all common in SUA [3, 4, 7, 19, 20]. A strong relationship exists between SUA and trisomy 13, trisomy 18, and triploid [7, 21], and the risk of chromosomal abnormalities is higher in foetuses with SUA and multiple structural anomalies than in foetuses with SUA and a single structural defect [21]. Furthermore, the influence of the missing side of the umbilical artery on foetal malformation shows conflicting results between studies [2, 22]. Guidelines recommend noting the number of umbilical cord vessels in the first trimester [23, 24]; this is optional in the second trimester depending on the feasibility of the technique and local practice [25]. Once SUA is detected, a thorough anatomical evaluation of the foetus is necessary. If no other abnormalities are observed, foetal echocardiogram and additional evaluation for aneuploidy are not recommended [1, 21, 26].

Many studies have demonstrated that SUA is highly correlated with fetal abnormalities, but have failed to describe the association of the SUA pattern with specific fetal malformations. A population-based retrospective study attempted to explain this pattern in Norway [7], although such studies are lacking in China. The present study was performed to assess the association between sonographically-identified SUA and different sonographically-identified structural malformations in singleton pregnancies in China, and to determine whether the missing side of the umbilical artery affected the outcomes. The results of this study can guide the focus of follow-up examinations in cases where SUA is detected during pregnancy.

## Methods

This was a retrospective study of singleton pregnancies in which routine anatomical screening was performed at the Obstetrics and Gynecology Hospital of Fudan University between January 2011 and April 2019 (*n* = 47,894). All procedures in this study were approved by the Human Research Ethics Committee of the Obstetrics and Gynecology Hospital, Fudan University.

### Prenatal screening

As a referral centre, the population included in this study comprised the general population for routine prenatal care and those referred in the first trimester for various reasons. All pregnant women in the general population underwent a detailed screening between 11^+ 0^ and 13^+ 6^ weeks of gestation to measure nuchal translucency thickness and detect major fetal abnormalities. Unless pregnancy termination was chosen, routine mid-trimester fetal ultrasound scans were performed between 20^+ 0^ and 24^+ 0^ weeks of gestation. Assessments of the absence of an umbilical artery and foetal anomalies by ultrasonography in both the first and second trimesters were mandatory. If abnormalities were first found during routine second-trimester screening, we performed a second detailed ultrasound to confirm and further evaluate the abnormalities for better pregnancy guidance. The detailed ultrasound included but was not limited to fetal echocardiography, and we performed this as early as possible within 28 weeks of gestation. Referrals who underwent routine anatomical screening at our centre were included in this study. If the pregnant woman was referred for abnormal findings in the routine first-trimester scan, we repeated the ultrasound; if the pregnancy continued, the second-trimester scan was performed routinely. If the patient was referred for other reasons, a routine anatomical scan was performed for the general population. Senior sonographers performed all examinations, and multidepartment consultations were performed for some abnormal foetuses.

### SUA diagnosis

**Fig. 1:**
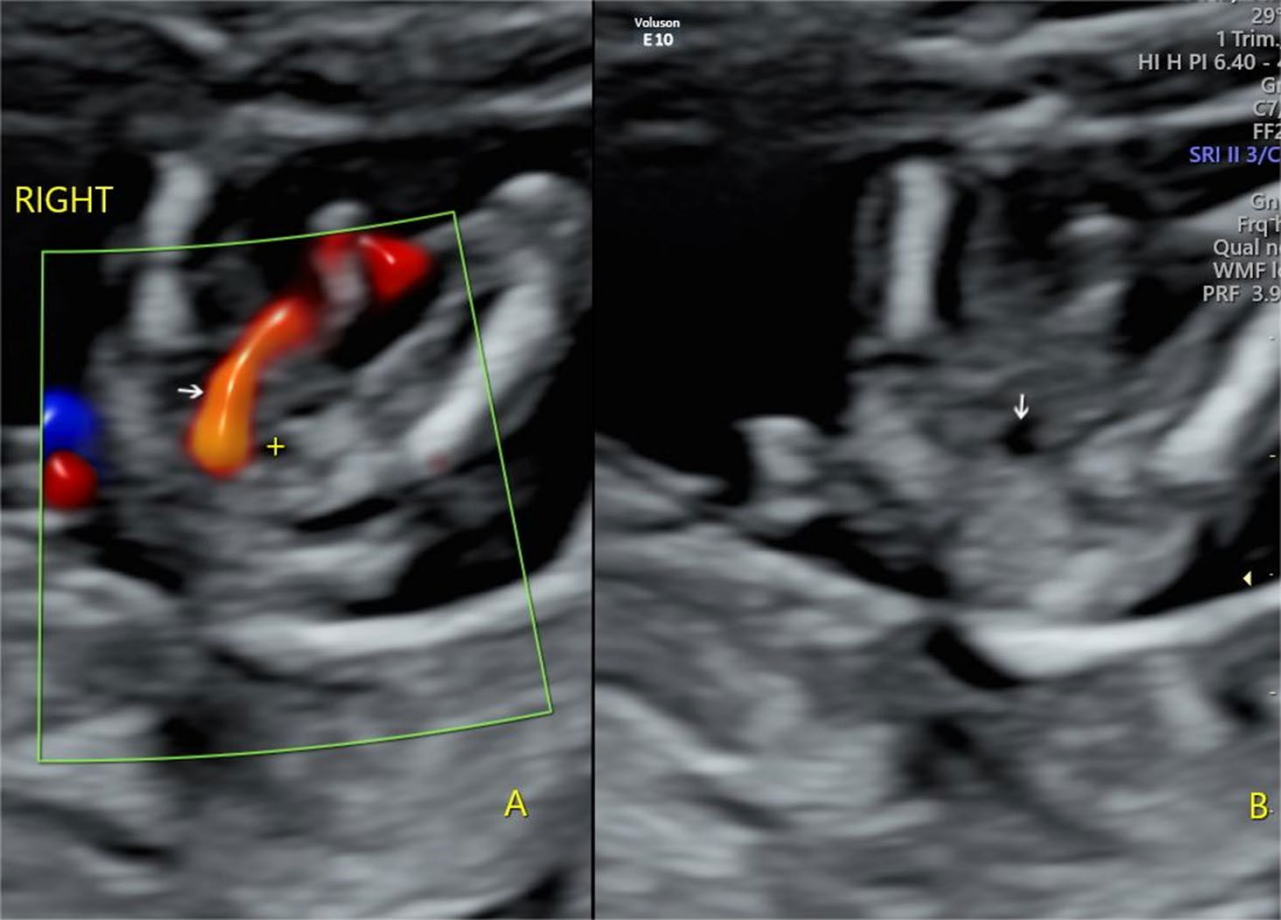
Axial view of foetal urinary bladder (12 + 6 weeks), showing (**A**) right single umbilical artery (arrow), urinary bladder (+) and (**B**) urinary bladder (arrow)

**Fig. 2:**
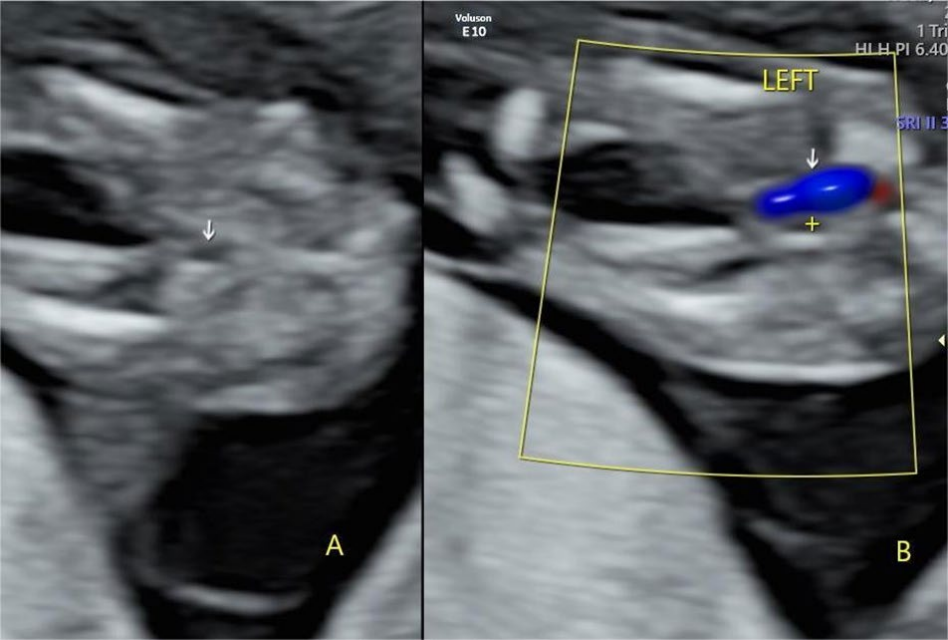
Axial view of foetal bladder (12 + 2 weeks), showing (**A**) urinary bladder (arrow) and (**B**) left single umbilical artery (arrow), urinary bladder (+)

SUA diagnosis was confirmed using two-dimensional images. As shown in Figs. 1 and 2, colour Doppler blood flow shows only one umbilical artery in the axial view of the foetal bladder and the umbilical artery on the other side is absent in cases of SUA; the umbilical cord cross-section reveals only one umbilical artery, whereas the colour Doppler blood flow signal shows one red and one blue. SUA was diagnosed in the first trimester screening using VOLUSON E8, E10, and 730 Expert (GE, Philadelphia, Pennsylvania, USA) and IU22 (Philips, Netherlands) ultrasound systems. SUA diagnosis was confirmed in the second trimester ultrasound screening, except for some pregnancies that were terminated voluntarily because of severe foetal abnormalities found in the first trimester and some foetal loss before the mid-trimester scan, including stillbirths and spontaneous abortions.

### Foetal abnormalities

Foetal abnormalities in different systems, including the craniocerebral, spinal, cardiovascular, gastrointestinal, genitourinary, limb and extremities, thoracic (except cardiovascular), anterior abdominal wall, face, neck, and others, were investigated and recorded.

We examined several common foetal malformations associated with SUA in our hospital, including craniocerebral malformations (cerebellar dysplasia, holoprosencephaly, dysgenesis of the corpus callosum, and encephalomeningocele), genitourinary (renal agenesis, multiple cystic hypoplastic, ectopic kidney, duplex kidney, and megalocystis), cleft lip and palate, gastrointestinal malformations (oesophageal atresia or stenosis, duodenal ileus, and small intestine obstruction), malformations of the abdominal wall (gastroschisis and omphalocele), chest deformities (diaphragmatic hernia and pulmonary dysplasia), malformations of the heart and great vessels (tetralogy of Fallot, ventricular septal defect, hypoplastic left heart, persistent left superior vena cava, right aortic arch, and double-outlet right ventricle), and deformities of the spine and limbs (spina bifida, scoliosis, and strephenopodia). All malformations diagnosed corresponded to the International Classification of Diseases 10 diagnoses Q0–99.

### Association between SUA diagnosis and foetal abnormalities

In our study, all foetuses with SUA diagnosed by ultrasound were divided into four groups: SUA combined with foetal abnormality, SUA combined with other soft ultrasound markers, SUA combined with abnormal pregnancy appendage, and the ISUA group. In the subgrouping, we defined the ISUA group as SUA without any other abnormal ultrasound findings, including soft ultrasound markers, to explore whether there were differences in which side the umbilical artery was missing when the ultrasonography results showed only SUA, SUA combined with soft markers, or SUA combined with malformations. The other soft ultrasound markers included: thickened nuchal translucency, absent or hypoplastic nasal bone, thickened nuchal fold, echogenic intracardiac focus, echogenic bowel, choroid plexus cysts, urinary tract dilation, and shortened humerus or femur or both, which were minor ultrasound findings in the first or second-trimester screening not representing a structural abnormality but associated with foetal anomalies [23, 26]. We further investigated whether an absent left or right umbilical artery was associated with a specific abnormality that was identified sonographically.

### Statistical analysis

Using logistic regression, odds ratios (OR) with 95% confidence intervals (CIs) were calculated for malformations associated with SUA. The Chi-square test was used to investigate whether there was a difference in which side the umbilical artery was absent in all the groups. All results were two-tailed, and statistical significance was set at *p* < 0.05. We used the Bonferroni method for pairwise comparisons between groups. Data analysis was performed using SPSS Statistics for Windows Version 22.0 (IBM Corp., Armonk, NY, USA).

## Results

Overall, 47,894 women with singleton pregnancies treated from January 2011 to April 2019 were deemed eligible for analysis, with 970 foetuses (2.0%) diagnosed with SUA in the first trimester by ultrasonography. Of this cohort, 463 (47.7%) foetuses presented with ISUA, while 387 (39.9 %) presented with SUA and additional abnormalities. In addition, there were 112 (11.5%) cases of SUA and other soft ultrasound markers, while 8 presented with SUA and pregnancy appendage abnormalities (including 5 cases of umbilical cord cyst, and 1 each of amniotic cyst, umbilical cord root parenchymal mass, and umbilical vein haemangioma).

Among all patients diagnosed with SUA complicated by foetal abnormalities using prenatal ultrasound, 223 (57.6%) presented with malformations in only 1 system, while 164 (42.4%) had malformations in at least 2 systems. Overall, the most common abnormalities were cardiovascular (50.6%) and genitourinary (22.2%). In foetuses with SUA and one system malformation, the most common was cardiovascular malformation (40.4%), followed by genitourinary (21.5%), gastrointestinal (7.2%), and craniocerebral (6.7%) malformations. The most common malformed system among those with multiple system malformations was the cardiovascular system (64.6%), followed by limb and extremity dysplasia (32.9%), facial (32.9%), cerebral (29.9%), and genitourinary (23.2%) systems. Table 1 shows the proportion of abnormalities in each system. All 23 infants with hygroma colli had oedema. Among the foetuses with multiple malformations, three, three, one, and one with abnormal cloaca, visceral inversion, thymus abnormality, and asplenia, respectively, were classified as having other abnormalities.

**Table 1:** Proportions of systemic malformations diagnosed by prenatal ultrasound in foetuses with a single umbilical artery

System	One system	≥ Two systems	Total
Craniocerebral	15 (6.7%)	49 (29.9%)	64 (16.5%)
Spinal	11 (4.9%)	30 (18.3%)	41 (10.6%)
Cardiovascular	90 (40.4%)	106 (64.6%)	196 (50.6%)
Gastrointestinal	16 (7.2%)	30 (18.3%)	46 (11.9%)
Genitourinary	48 (21.5%)	38 (23.2%)	86 (22.2%)
Limb and extremities	9 (4.0%)	54 (32.9%)	63 (16.3%)
Thorax	4 (1.8%)	19 (11.6%)	23 (5.9%)
Anterior abdominal wall	12 (5.4%)	22 (13.4%)	34 (8.8%)
Face	10 (4.5%)	54 (32.9%)	64 (16.5%)
Neck	8 (3.6%)	15 (9.1%)	23 (5.9%)
Others		8 (4.9%)	8 (2.1%)

Table 2 shows the association between SUA and specific malformations. A robust association was observed between SUA and oesophageal stenosis or atresia (OR: 25.33), followed by cardiovascular malformations (OR: 9.98–24.02), scoliosis (OR: 18.62), and genitourinary malformations (OR: 2.45–15.66). We further identified a strong association between SUA and brain malformations (OR: 4.73–9.12). In addition, we found no difference in the risk of gastroschisis and duplex kidneys between foetuses with SUA and foetuses with three umbilical vessels.

**Table 2:** Risk of specific foetal malformations with SUA and the association with the absent side

Outcome	SUA		OR (95%CIs)	Absent side (left/right/no record)	*p*-value
	YES	NO			
Tetralogy of Fallot	24	72	16.51(10.36–26.32)	11/12/1	0.76
Ventricular septal defect	55	230	12.20(9.03–16.50)	25/24/6	0.99
Hypoplastic left heart	17	75	11.14(6.56–18.94)	6/6/5	0.91
Persistent left superior vena cava	45	95	23.98(16.72–34.39)	23/17/5	0.39
Right aortic arch	9	44	9.98(4.86–20.50)	4/5/0	0.75
Double-outlet right ventricle	19	39	24.02(13.83–41.72)	12/4/3	0.06
Omphalocele	23	122	9.32(5.94–14.62)	10/12/1	0.59
Gastroschisis	2	36	2.69(0.65–11.19)	0/0/2	
Renal agenesis	28	93	14.97(9.76–22.95)	11/16/1	0.31
Multiple cystic hypoplastic	15	109	6.75(3.92–11.62)	7/7/1	0.94
Ectopic kidney	7	87	3.92(1.81–8.47)	6/1/0	0.10
Duplex kidney	5	99	2.45(0.99–6.03)	4/1/0	0.23
Megalocystis	9	28	15.66(7.38–33.33)	4/3/2	0.74
Oesophageal atresia or stenosis	28	55	25.33(16.99–40.12)	12/13/3	0.86
Duodenal ileus or small intestine obstruction	5	87	2.79(1.13–6.89)	2/3/0	0.61
Diaphragmatic hernia	9	96	4.57(2.30–9.07)	3/5/1	0.46
Pulmonary dysplasia	5	25	9.72(3.71–25.44)	0/4/1	0.99
Cleft lip and palate	23	416	2.72(1.78–4.15)	9/11/3	0.60
Cerebellum dysplasia	18	123	7.19(4.37–11.85)	7/7/4	0.97
Holoprosencephaly	7	72	4.73(2.17–10.30)	2/3/2	0.61
Dysgenesis of the corpus callosum	8	81	4.81(2.32–9.97)	4/3/1	0.73
Encephalomeningocele	6	32	9.12(3.81–21.86)	1/3/2	0.31
Spina bifida	6	61	4.78(2.06–11.09)	2/4/0	0.44
Scoliosis	20	53	18.62(11.09–31.26)	8/11/1	0.46
Strephenopodia	19	179	5.22(3.24–8.41)	10/6/3	0.37

The total sample size for no SUA was 46,924 and for yes SUA was 970;

Furthermore, we found that the absence of the left umbilical artery (*n* = 445, 45.9%) was consistent with that of the right umbilical artery (*n* = 431, 44.4%). However, as shown in Table 3, the side of the absent umbilical artery was significantly different between the ISUA and SUA with foetal abnormalities groups (*p*<0.01). In foetuses with SUA and malformations, the right umbilical artery had a higher rate of absence than the left. However, there was no statistically significant difference in which side the umbilical artery was absent between the ISUA and SUA with other soft ultrasound markers groups (*p* = 0.62). The absence of a right or left umbilical artery did not affect any specific foetal abnormality in this analysis (Table 2).

**Table 3:** Differences in the absent side of the umbilical artery between the ISUA group and other groups

Group	Left	Right	No record	χ^2^	*p*-value
ISUA	235	189	39		
SUA with other soft ultrasound markers	52	48	12	0.96	0.62
SUA with foetal anomalies	155	189	43	9.87	<0.01
SUA combined with abnormal pregnancy appendage	3	5	0		

ISUA: isolated single umbilical artery; SUA: single umbilical artery

χ2, Chi-square test;

Table 4 shows the pregnancy outcomes of women with SUA, including 405 cases of live births, 83 voluntary termination of pregnancy after discovering foetal abnormalities, 16 stillbirths, 1 spontaneous abortion, and 463 lacking data.

**Table 4:** Pregnancy outcomes of SUA cases

Group	Live birth	Abandonment of pregnancy	Intrauterine death	Spontaneous abortion	Lack of data
ISUA	315	5	4	1	138
SUA with other soft ultrasound markers	44	2	4	/	62
SUA with foetal anomalies (one system)	43	36	5	/	139
SUA with foetal anomalies (≥ two systems)	/	42	3	/	119
SUA combined with abnormal pregnancy appendage	3	/	/	/	5
Total	405	85	16	1	463

ISUA: isolated single umbilical artery; SUA: single umbilical artery

In 315 cases of ISUA who had a live birth, the mean age of the women was 30.9 (± 4.1) years, 53 (16.8%) had gestational diabetes mellitus, 21 (6.7%) had pregnancy-induced hypertension; 155 (49.2%) neonates were delivered vaginally, while 160 (50.8%) were delivered by caesarean section. The average gestational age at delivery was 38.4 (± 1.6) weeks, and 29 (9.2%) were preterm. The mean neonatal birth weight was 3167.6 (± 541.4) g, and the neonatal 1-min Apgar score was 8.9 (± 0.6).

In 315 cases of ISUA neonates, 1 each of left ear vegetation and left jaw haemangioma were found by routine examination of appearance at birth; 1 case of neonatal congenital intestinal atresia or stenosis was considered after examination owing to abnormal feeding. Of the 315 neonates, 55 underwent imaging examination at our hospital, and some abnormal findings were found, as shown in Table 5. Among 44 live births of foetuses with SUA with other soft markers, 1 was diagnosed with hypospadias by routine postpartum examination and 6 by cardiac ultrasound. The results are shown in Table 5.

**Table 5:** Imaging findings of a few newborns with a single umbilical artery

	ISUA			SUA with other soft ultrasound markers		
Examination item	Number of examination	Abnormal findings and number		Number of examinations	Abnormal findings and number	
Cardiac ultrasound	54	Atrial septal defect	5	6	Atrial septal defect, ventricular septal defect and small right ventricle	1
		Ventricular septal defect	2		Mild tricuspid regurgitation	2
		Mild tricuspid regurgitation	14			
		Tricuspid regurgitation with pulmonary hypertension	3			
		Mitral regurgitation	4			
		Pericardial effusion	1			
		Persistent left superior vena cava	1			
Cranial ultrasound	16	Intracranial haemorrhage	3	1	/	
		Intracranial cystic foci	3			
		Partial absence of septa pellucida	1			
Abdominal and urinary system ultrasound	46	Hepatic vascular malformation	1	1	/	
Spinal X-ray	17	Scoliosis	4			
		Abnormal lumbar spine body	1			

ISUA: isolated single umbilical artery; SUA: single umbilical artery

## Discussion

A strong relationship between SUA and foetal malformations has been previously reported in the literature [3]. Several reports [4, 5, 7, 19] have shown that approximately 10.9–28.3% of SUA cases were associated with at least one malformation. In the present study, 39.9% of foetuses with SUA had structural malformations; the most common abnormalities were cardiovascular (50.6%) and genitourinary (22.2%) abnormalities, which is consistent with the findings reported by Hua et al. and Friebe-Hoffmann et al. [19, 20], but conflicts with other studies [3, 4] which suggested that genitourinary malformations may be most common. The percentage of SUA cases with anomalies in this study was higher than that reported in previous studies. One possible explanation involves the source of our data. As a renowned specialised hospital in China, the Obstetrics and Gynecology Hospital of Fudan University has a strong prenatal diagnosis team; as such, the number of pregnant women with abnormalities who present for further examination is high.

Ebbing et al. [7] showed a pattern of congenital malformations in foetuses with SUA and neonates in Norway and an especially strong association was observed between SUA and oesophageal atresia (OR: 25.82), cardiovascular (OR: 2.86–7.62), renal agenesis (OR: 5.94), diaphragmatic hernia (OR: 4.78), and limb reduction (OR: 4.61). In the present study, our results further supported the association between SUA and oesophageal atresia or stenosis (OR: 25.33), as well as diaphragmatic hernia (OR: 4.57). Owing to the limitations of data collection, we described the relationship between SUA and a few specific foetal malformations rather than a class of malformations, which may have led to the large ranges of OR values between SUA and congenital malformations in our study, especially in the cardiovascular (OR: 9.98–24.02) and genitourinary (OR: 2.45–15.66) systems. Regarding renal anomalies, we found a strong association between SUA and megacystis (OR: 15.66), which is the main cause of lower urinary tract obstruction [27]. As SUA has a high association with foetal cardiovascular anomalies, whether foetal echocardiography is needed during pregnancy still needs to be evaluated. Several studies [28–30] have previously shown that if the detailed ultrasonographic anatomic survey of the foetuses with SUA is negative, foetal echocardiography is unnecessary unless the routine anatomic scan does not include an appropriate heart examination [31]. However, foetal echocardiography is commonly recommended for foetuses with SUA and extracardiac abnormalities or maternal risk factors for congenital heart disease [32]. A previous study recommends routine physical examinations for newborns with ISUA and considers additional examinations unnecessary [33]. In this study, we found several cases of abnormalities in postnatal echocardiography, inconsistent with prenatal abnormalities, most of which were atrial septal defects or ventricular septal defects; we also noted some new findings in other examinations. Perhaps parents can decide whether further examinations should be conducted on the newborn.

In the present study, we found that the absence of the left umbilical artery (*n* = 445, 45.9%) was consistent with that of the right umbilical artery (*n* = 431, 44.4%), although right umbilical artery deletion was more common in SUA with foetal abnormalities (*p*<0.01). This result differs from the findings of Wu et al., who revealed no difference in which side umbilical artery was absent in foetuses with SUA and anomalies [2]. However, the observed differences between the absent side of the umbilical artery and each specific anomaly in this study were not statistically significant. Overall, 94 foetuses (9.7%) had a missing umbilical artery, the details of which were not recorded, which may have influenced the results. Furthermore, the small sample size for each malformation may have skewed our results.

One major strength of this study is that the study population included all mothers who underwent routine screening for abnormalities in the first and/or second trimesters, thereby minimising the risk of selection bias and ensuring the accuracy of prenatal ultrasound diagnosis to the greatest extent possible. Another strength is that we assessed the occurrence of different malformation types in SUA, including single-system and multisystem malformations, and attempted to decipher the association of SUA with specific foetal malformations, which may have a certain reference significance for prenatal examination. We further sought to determine whether SUA combined with other soft ultrasound indicators and ISUA would differ based on which side the umbilical artery was missing. In addition, we recorded the postnatal physical examination and partial additional examination results for SUA newborns who did not show structural abnormalities prenatally. Most of the newborns exhibited normal findings, although a few cases with structural abnormalities were discovered after birth. Performing additional examinations on newborns with prenatal indications of ISUA may be considered an option.

Nevertheless, this study has a few limitations. First, as a referral centre, the population included in this study comprised the general population for routine prenatal care and referrals for further examination; this may have biased the selection process and be related to the high malformation rate in foetuses with SUA. However, as a retrospective study, completely distinguishing between the general and referred populations was difficult. Additionally, we accepted patients for various reasons, not restricting to pregnant women with single umbilical artery and foetal abnormalities, which could dilute the high-risk population resulting from referral to a certain extent. Second, SUA and foetal malformations were diagnosed using prenatal ultrasound; however, some of the data on umbilical cord records, foetal autopsies, or neonatal examinations after termination were missing, and an assessment of the accuracy of prenatal diagnosis for SUA and foetal structural abnormalities was not possible. As shown in previous studies, the detection rate of foetal malformations in the first trimester can reach more than 60% [34], with a high positive predictive value (94.7%), and a high negative predictive value (98.7%) [35]. Compared with the records following pregnancy termination, the sensitivity, specificity, and accuracy of the mid-trimester ultrasound screening for congenital malformation detection were 61.1%, 96.3%, and 94.3%, respectively [36]. The detection rate can be as high as 81.4% before the third trimester [37]. In addition, if more factors, such as smoking history, alcohol use, alcohol abuse history, exposure to harmful substances, and adverse maternal history, were included in the study, a better explanation of the association between SUA and foetal anomalies could be provided. However, we conducted this study to assess the relationship between SUA and specific anomalies that could be found by prenatal ultrasound screening, rather than genetic syndromes, which may involve a variety of malformations. Furthermore, this study did not record foetal karyotypes and genes and did not discuss the relationship between SUA and foetal chromosome and gene abnormalities.

## Conclusions

In summary, we found that SUA was strongly associated with foetal malformations in multiple systems, most commonly the cardiovascular system. Further, we found a strong association between SUA and oesophageal stenosis or atresia. The absence of the right umbilical artery was more common in foetuses with SUA and structural malformations. To better describe the pattern and provide improved guidance for the examination of foetuses with SUA, further studies using only the general population, a larger sample, and information on more maternal factors, foetal chromosomes, genes, foetal autopsy, and postnatal examinations, are necessary.

## Data Availability

The datasets generated and/or analysed during the current study are not publicly available due to patient privacy but are available from the corresponding author on reasonable request.

## Abbreviations

SUA: single umbilical artery
ISUA: isolated single umbilical artery
NICU: neonatal intensive care unit
OR: odds ratios
CIs: confidence intervals
